# Retrospective molecular analyses of hard ticks (Acari: Ixodidae) from patients admitted to the Centre for Tick-Borne Diseases in Central Europe, Hungary (1999–2021), in relation to clinical symptoms

**DOI:** 10.1186/s13071-025-06880-2

**Published:** 2025-06-20

**Authors:** Sándor Hornok, Nóra Takács, Gyöngyi Nagy, András Lakos

**Affiliations:** 1 Department of Parasitology and Zoology, University of Veterinary Medicine, Budapest, Hungary; 2 Hungarian Research Network (HUN-REN)-University of Veterinary Medicine Budapest (UVMB) Climate Change: New Blood-Sucking Parasites and Vector-Borne Pathogens Research Group, Budapest, Hungary; 3 Centre for Tick-Borne Diseases, Budapest, Hungary

## Abstract

**Background:**

This study aimed at investigating the diversity of pathogens in human-biting ixodid ticks, in relation to their seasonality and associated clinical symptoms.

**Methods:**

Hard ticks, collected from humans in the course of 23 years, were identified to the species level on a morphological basis. This was followed by DNA extraction and molecular analyses. The latter served to confirm tick species, and to detect important tick-borne pathogens, in particular rickettsiae, Anaplasmataceae, borreliae, and piroplasms.

**Results:**

Among 502 ticks, six species were identified, with the predominance of *Ixodes ricinus*. Considering tick-borne pathogens, four *Rickettsia* spp., *Anaplasma phagocytophilum*, seven genospecies of *Borrelia burgdorferi sensu lato*, and three *Babesia* spp. were detected. Some of these predominated in nymphs or females of *I. ricinus*. Tick-infested patients presented with six types of clinical signs. Approximately one out of seven ticks from patients presenting with erythema migrans were unengorged. Shorter, spring-associated presence of *Babesia microti*-, *A. phagocytophilum*-, and *Dermacentor*-borne rickettsiae was observed in ticks, while *Rickettsia helvetica* and borreliae persisted until late autumn.

**Conclusions:**

The seasonal occurrence of *I. ricinus*-borne pathogens appeared to be genus-dependent, but did not correlate with known typical reservoirs (rodents, birds, reptiles), nor with tick developmental stage or transstadial versus transovarial transmission. Pathogen detection in ticks that bit humans did not necessarily imply an infection.

**Graphical Abstract:**

**Figure:**
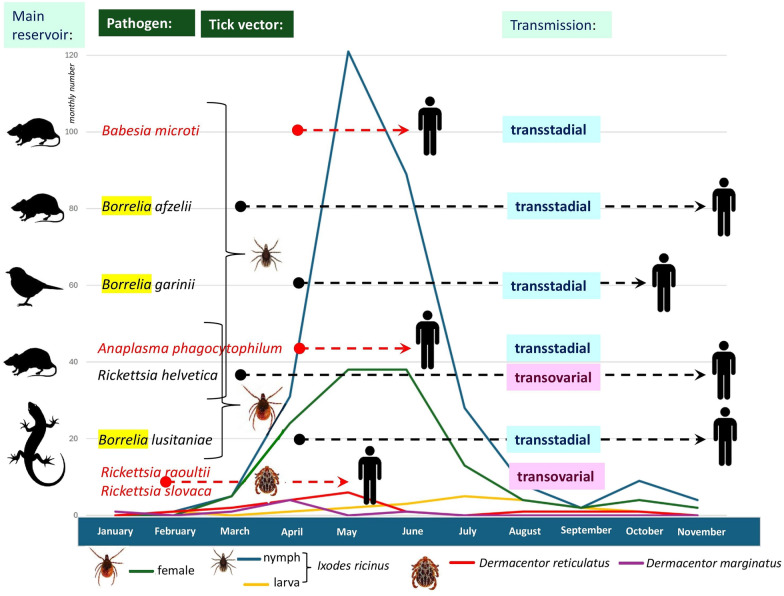

**Supplementary Information:**

The online version contains supplementary material available at 10.1186/s13071-025-06880-2.

## Background

Among hard ticks (Acari: Ixodidae), nearly 40% of species can infest humans, and most of these are potential carriers of zoonotic pathogens [1]. In total, tick-borne pathogens are responsible for more than 100,000 annual cases of illnesses in humans throughout the world [2]. In Europe, nowadays, the incidence and public health burden of tick-borne pathogens tend to intensify due to various factors, including anthropogenic ones [3] and climate change [4].

Zoonotic pathogens usually have their main reservoirs among wild living vertebrates, as exemplified by rodents, birds, and reptiles in the case of the most prevalent group of tick-borne pathogens of the northern hemisphere: *Borrelia burgdorferi sensu lato* [3]. This group consists of the causative agents of Lyme disease in North America [5], Asia [6] and Europe [7]. In Europe, the overall mean prevalence of *B. burgdorferi* genospecies in ticks has been estimated at about 12%, with higher prevalence in adult ticks than nymphs. On this continent, Central Europe is the region with the highest tick infection rates (nymphs > 10%; adult ticks > 20%), in particular Austria, Czech Republic, southern Germany, Switzerland, Slovakia, and Slovenia [7]. While Hungary is not listed above, the prevalence of Lyme borreliae in their main vector, *Ixodes ricinus*, was reported to be up to 50% in urban habitats [8]. In this country, an additional three tick species were reported to infest humans, i.e., *Alloceraea* (formerly *Haemaphysalis*) *inermis*, *Dermacentor reticulatus*, and *Dermacentor marginatus* [9]. The latter two species may transmit rickettsiae that are the causative agents of tick-borne lymphadenopathy (TIBOLA) in humans, as reported in Hungary for the first time in a worldwide context [10].

However, there was no comprehensive survey on human-biting ticks and the pathogens they might carry in Hungary. The aim of this study was to compensate for this lack of information, by molecularly analyzing ticks that were removed from humans admitted to the Centre for Tick-Borne Diseases in Budapest during the past decades.

## Methods

### Origin and identification of samples

Ticks were collected from human subjects (*n* = 487; the sex recorded in the majority of cases was 220 male and 261 female subjects), who sought medical care after a tick bite, at the Centre for Tick-Borne Diseases in Budapest, Hungary, between 1999 and 2021. Via the Internet and popular media, we encouraged people who were bitten by ticks to preserve the parasite during this period. Ticks were randomly stored either dried (under Scotch tape on paper) or soaked in 70% ethanol in 1.5 mL screw-cap tubes. The oldest samples stored dry (on paper) with successful tick DNA and pathogen DNA detection date back to 1999 and 2002; i.e., they were preserved for 24 and 21 years, respectively. Prior to DNA extraction carried out in 2023–2024, the species of ticks were identified on the basis of standard identification keys [11]. Most ticks (*n* = 464) could also be assigned to four groups according to their estimated degree of engorgement as apparently unengorged (corresponding to approximately day 1 of attachment), as slightly engorged (after approximately 2 days), as moderately engorged (around the third day) and as close to full engorgement (after approximately 4 days of attachment), as shown in Additional File 1 (Fig. S1), based on Gray et al. [12].

### Molecular and phylogenetic analyses

DNA was extracted from whole ticks with the QIAamp DNA Mini Kit (QIAGEN, Hilden, Germany), including an overnight digestion in tissue lysis buffer and Proteinase K at 56 °C. To assess the success of DNA extraction, first the polymerase chain reaction (PCR) amplification of an approximately 460-bp fragment of the *16S rRNA* gene of Ixodidae was attempted, with the primers 16S + 1 (5′-CTG CTC AAT GAT TTT TTA AAT TGC TGT GG-3′) and 16S-1 (5′-CCG GTC TGA ACT CAG ATC AAG T-3′). The reaction mixture contained 1 U (0.2 µL) HotStarTaq Plus DNA polymerase, 2.5 µL 10 × CoralLoad Reaction buffer (with 15 mM MgCl_2_), 0.5 µL PCR nucleotide mix (0.2 mM each), 0.5 µL (1 µM final concentration) of each primer, 15.8 µL double distilled water (ddH_2_O) and 5 µL template DNA in a volume of 25 µL. The PCR included an initial denaturation step at 95 °C for 5 min, followed by 40 cycles of denaturation at 94 °C for 40 s, annealing at 51 °C for 1 min and extension at 72 °C for 1 min. Final extension was done at 72 °C for 10 min. PCR products of this PCR were only sequenced in selected cases, if confirmation of morphological identification of tick species was necessary (e.g., if they were severely damaged). This PCR was also used to judge the success of DNA extraction from ticks stored in different ways. There was no significant difference between the rate of PCR positivity among samples dried (on paper) or stored in ethanol (data not shown).

The PCR on the *16S rRNA* gene of Ixodidae was followed by screening of pathogens in all samples and confirming *Borrelia* genospecies identity at least once for each genospecies with the methods summarized in Additional File 2 (Table S1). Reaction components in the pathogen screening conventional PCRs were the same as in the above *16S rRNA* gene test, except using 14.8 µL DW and 1.0 µL of both primers (10 μM stock, final concentration 0.4 μM) in the *Borrelia* PCR mix, and 2.5 µL DNA template with 18.3 µL double-distilled water in the *Rickettsia* PCR mix. Reaction components of the real-time PCR screening *Anaplasma phagocytophilum* were as follows: total volume 10 µL, containing 2.5 µL DNA template, 1.8 µL DW, 5 µL KAPA Probe Fast qPCR Mastermix (2X) (Kapa Biosystems Inc., Wilmington, USA), 0.3 µL both primers (10 μM stock; final concentration 0.3 μM) and 0.1 µL probe (10 μM stock).

Purification and sequencing of the PCR products were done by Eurofins Biomi Ltd. (Gödöllő, Hungary). Quality control and trimming of sequences were performed with the BioEdit program. Obtained sequences were compared with GenBank data by the nucleotide BLASTN program (https://blast.ncbi.nlm.nih.gov). New sequences were submitted to GenBank under the following accession numbers: *Babesia* sp. *18S rRNA* gene: PQ764142-PQ764144, other pathogens: PQ773226-PQ773269. During phylogenetic analyses of *Borrelia* genospecies, sequence datasets were resampled 1000 times to generate bootstrap values. Phylogenetic analysis was performed with the Neighbor-Joining method, p-distance model in MEGA7.

### Evaluation of clinical signs, serological follow-up, and assignment of patients to disease group

The Hungarian Lyme disease guideline recommends stricter than regularly accepted rules for diagnosing erythema migrans (EM). We only accept a diagnosis of EM if the red spot in the case of a recognized tick bite exceeds 5 cm in diameter and, moreover, it spreads and increases in size for 3 more days. The daily increase of 0.5–2 cm in diameter is also an important diagnostic parameter for EM. If the skin reaction does not fulfill these criteria, we consider the reaction to be a foreign body reaction, registered as “dermatitis.” If the erythema spreads rapidly and becomes painful and itchy, we consider this inflammation to be an allergic reaction to the saliva or other component of the tick. These patients were also enrolled in the “dermatitis” group. Serological status was monitored using paired serum samples drawn at an interval of at least 3 weeks in tick-bitten female patients, who were pregnant, in order to exclude infection or seroconversion [13].

The most important clinical symptoms of tick-borne lymphadenopathy (TIBOLA) were first described in 1997 by the senior author of this study [10]. This infection is caused by *Rickettsia slovaca* or *Rickettsia raoultii*, transmitted by *Dermacentor* sp. ticks. The characteristic symptoms are an eschar with a honey-like discharge on the scalp with enlarged lymph nodes behind the sternocleidoid muscle [14]. In addition, the diagnosis of *Borrelia* lymphocytoma (BL) was based on the appearance of a purple, swollen, painless, non-itching lesion, particularly on the earlobe of children (sometimes involving the whole ear pinna) and on the nipple in adults. If no relevant clinical signs were noted, the patient was assigned to the *sine morbo* (SM) group.

Whenever conditions were appropriate, tick providers were asked to indicate the likely origin of location of their tick exposure (i.e., garden, excursion into a forest, etc.). Altogether 157 patients provided information on this. In addition, whenever known, the sex of patients was analyzed in the context of tick-borne pathogens (Additional file 4: Table S2), and two age groups (children: up to 18 years old versus adults) were compared between patients presenting with TIBOLA or EM.

### Statistical analyses

Ticks carried on host from other Northern- or Central-European countries into Hungary (*n* = 8) were excluded from seasonal analyses. Prevalence data were compared using Fisher’s exact test (https://www.langsrud.com/fisher.htm) and differences were regarded as significant if *P* < 0.05. Confidence intervals (CIs) for prevalence rates were calculated at the level of 95%.

## Results

### Species, developmental stages, and spatiotemporal occurrence of human-infesting ticks

In this study, 502 human-infesting ticks were used, 494 of which originated from Hungary (the others were found on patients traveling from Sweden, Finland, Germany, Switzerland, Czechia, Austria, Slovakia, and Romania). Six ixodid tick species were identified, among which *Ixodes ricinus* predominated (*n* = 466; 92.8%; 95% CI 90.2–94.9%), followed in decreasing order by *Dermacentor reticulatus* (*n* = 22; 4.4%; 95% CI 2.8–6.6%), *Dermacentor marginatus* (*n* = 9; 1.8%; 95% CI 0.8–3.4%), *Ixodes hexagonus* (*n* = 2; 0.4%; 95% CI 0.1–1.4%), *Haemaphysalis concinna* (*n* = 2; 0.4%; 95% CI 0.1–1.4%), and *A. inermis* (*n* = 1; 0.2%; 95% CI 0.01–1.1%). The majority (312 of 466; 67%; 95% CI 62.5–71.2%) of *I. ricinus* specimens were nymphs, and among developmental stages of this tick species, larvae had the lowest ratio (20 of 466; 4.3%; 95% CI 2.6–6.6%) (Table 1). Importantly, a single larva or nymph of three additional species, *D. reticulatus*, *D. marginatus*, and *H. concinna*, were also found to infest humans. However, *I. hexagonus* and *A. inermis* were only represented by adults. All male ticks collected in this study from humans belonged to the genus *Dermacentor* (Table 1).

**Table 1:** Number of human-infesting ticks collected in this study, according to their species, developmental stage, or sex

Tick species	Sex or stage (*n*)	Detected pathogens												
		BOGA	BOBA	BOLU	BOVA	BOBU	BOAF	BOSP	RIHE	RIMO	RIRA	RISL	ANPH	*Babesia* (*n*)
*Ixodes ricinus*	Larva (20)									1			1	
	Nymph (312)	5	2			2	45	4	25	6			8	BAMI (8) BACA (1)
	Female (134)	1		5	2	1	8		13	6			9	BAMI (1)
*Ixodes hexagonus*	Female (2)													
*Dermacentor reticulatus*	Larva (1)													
	Male (3)													
	Female (18)										9			
*Dermacentor marginatus*	Nymph (1)													
	Male (2)										1			
	Female (6)											2		
*Haemaphysalis concinna*	Nymph (1)													
	Female (1)													BAIRK (1)
*Alloceraea inermis*	Female (1)													
In total:	502	6	2	5	2	3	53	4	38	13	10	2	18	11

The number ticks in which pathogens were molecularly detected is shown according to the abbreviation of the genus (first two letters) and species name (first two letters) of tick-borne pathogen as outlined in the table notes below

*BOGA*, *Borrelia garinii*; *BOBA*, *Borrelia bavariensis*; *BOLU*, *Borrelia lusitaniae*; *BOBU*, *Borrelia burgdorferi *sensu stricto; *BOAF*, *Borrelia afzelii*; *BOSP*, *Borrelia spielmanii*; *RIHE*, *Rickettsia helvetica*; *RIMO*, *Rickettsia monacensis*; *RIRA*, *Rickettsia raoultii*; *RISLO*, *Rickettsia slovaca*; *ANPH*, *Anaplasma phagocytophilum*; *BAMI*, *Babesia microti*; *BACA*, *Babesia capreoli*; *BAIRK*, *Babesia* sp. Irkutsk

Based on answers to inquiries on the origin of tick bite, the majority (70 of 157; 44.6%; 95% CI 36.7–52.7%) of patients indicated tick exposure in their own or their relative’s garden, and only 15.9% (25 of 157; 95% CI 10.6–22.6%) mentioned an excursion, with the remaining possibilities being much rarer (e.g., during collection of fungi, fishing, honeybee keeping, hunting, work, or sport).

**Fig. 1:**
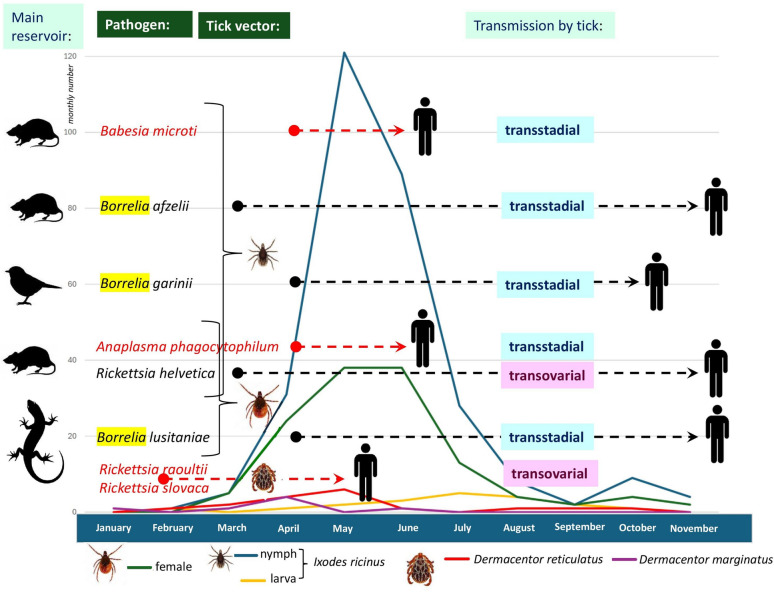
Monthly number of most often collected human-infesting ticks according to their species and (in the case of *Ixodes ricinus*) developmental stages. In addition, the dashed lines indicate the months during which a pathogen was detected in ticks removed from humans. A red dashed line marks those periods which were restricted to the first half of the year (i.e., spring tick season). According to these tick-borne pathogens, the silhouette of most likely reservoirs (rodents, birds, or reptiles; based on literature data), the predominant carrier in this study (nymphs or females or both in the case of *I. ricinus* and *Dermacentor reticulatus*), as well as typical modes of transmission (transstadial versus transovarial, based on literature data) are also shown. Curly brackets connect pathogens that were demonstrated predominantly from either nymphs or females of *I. ricinus*

Considering seasonality according to species, the highest numbers of *D. marginatus* and *D. reticulatus* were collected in April and May, respectively. Among *I. ricinus* specimens infesting humans, nymphs had an activity peak in May and females in May and June, whereas larvae had an activity peak in July (Fig. 1). A single nymph of *H. concinna* was removed from its host in June, and the female of this species in July. In addition, the female of *A. inermis* was found on a human subject in April, in addition to one *I. hexagonus* female in May and another in November.

### Species, seasonality, and distribution of tick-borne pathogens according to tick species and developmental stages

In general, pathogen DNA was detected in 28.7% of ticks (144 of 502; 95% CI 24.8–32.9%). In particular, a higher ratio of *Dermacentor* spp. (12 of 31; 38.7%; 95% CI 21.9–57.8%) than *I. ricinus* (131 of 466; 28.1%; 95% CI 24.1–32.4%) were found infected. Multiple pathogens were only present in *I. ricinus*, and those always belonged to different genera. Altogether, 15.2% of *I. ricinus* (75 of 466; 95% CI 12.9–19.8%) contained the DNA of *Borrelia burgdorferi s. l.*, 8.2% (38 of 466; 95% CI 5.8–11%) that of *Rickettsia helvetica*, followed in decreasing order by *A. phagocytophilum* (18 of 466; 3.9%; 95% CI 2.3–6%), *Rickettsia monacensis* (13 of 466; 2.8%; 95% CI 1.5–4.7%), *Babesia microti* (9 of 466; 1.9%; 95% CI 0.9–3.6%), and a single case of *Babesia capreoli* (Table 1). In *Dermacentor* spp., *Rickettsia raoultii* was significantly (*P* = 0.02) more frequently detected (10 of 31; 32.3%; 95% CI 16.7–51.4%), than *Rickettsia slovaca* (2 of 31; 6.5%; 95% CI 0.8–21.4%).

*Borrelia burgdorferi s. l.* was represented by seven genospecies, including (in decreasing order of frequency) *Borrelia afzelii* (53 of 75; 70.7%; 95% CI 59–80.6%), *Borrelia garinii* (6 of 75; 8%; 95% CI 3–16.6%), *Borrelia lusitaniae* (5 of 75; 6.7%; 95% CI 2.2–14.9%), *Borrelia spielmanii* (4 of 75, 5.3%; 95% CI 1.5–13.1%), *Borrelia burgdorferi *sensu stricto (3 of 75; 4%; 95% CI 0.8–11.3%), and *Borrelia valaisiana* (2 of 75; 2.7%; 95% CI 0.3–9.3%), *Borrelia bavariensis* (2 of 75; 2.7%; 95% CI 0.3–9.3%). Phylogenetically, *B. afzelii* belonged to two groups (group B: 38 ticks; group A: 11 ticks) (Additional file 3: Fig. S2). Although *B. afzelii* group A DNA was not detected in female ticks, only in nymphs (*n* = 12), this was a non-significant association (group B was present in 8 female ticks and 34 nymphs). The seasonal occurrence of *Borrelia* genospecies in human-infesting ticks was concentrated in the period from April to June (Fig. 1; Additional file 4: Table S2).

Among other tick-borne pathogens associated with *I. ricinus*, the DNA of *R. helvetica* and *R. monacensis* were shown in ticks from early spring (March) to late autumn (November). This was different from *A. phagocytophilum* and piroplasm (*B. microti* and *B. capreoli*) carrier *I. ricinus* ticks, which had a narrow temporal distribution, in April, May, and June, meaning a significant association with the latter period (*P* = 0.013) (Fig. 1; Additional file 4: Table S2).

*Dermacentor*-associated rickettsiae, i.e., *R. raoultii* and *R. slovaca* were detected in ticks collected from late winter (February) to early summer (June) (Fig. 1; Additional file 4: Table S2). *Babesia* sp. Irkutsk was present in a single *H. concinna* female in July.

Regarding pathogen burdens according to developmental stages and sexes of ticks, *B. burgdorferi s. l.* was not detected in *I. ricinus* larvae. *Borrelia lusitaniae* was significantly associated with female *I. ricinus* (*P* = 0.0023; 0 of 312 nymphs versus 5 of 134 females infected) but *B. afzelii* with nymphs (*P* = 0.01; 45 of 312 nymphs versus 8 of 134 females infected). This means a significant difference between the exclusive presence of *B. lusitaniae* in females of human-biting *I. ricinus*, in contrast to the predominance of both *B. garinii* and *B. afzelii* in nymphs of this tick species (*P* = 0.015 and *P* = 0.00028, respectively) (Table 1). The DNA of *R. raoultii* and *R. slovaca* were almost exclusively detected in females of the genus *Dermacentor*, but the former rickettsia was also present in a male *D. marginatus* (Table 1). *Anaplasma phagocytophilum* was found in nymphs and females of *I. ricinus*, but also in a single larva. Among piroplasms, although all except one infection of *I. ricinus* with *B. microti* was detected in nymphs (*n* = 8), this was a non-significant association (Table 1).

The prevalence of tick-borne pathogens was not significantly associated with the sex of human hosts (calculation not shown; Additional file 4: Table S2).

### Clinical relevance of detected pathogens

Among pregnant female patients with serological-clinical follow-up, 24 had no relevant clinical signs or seroconversion, but pathogens were detected in the ticks removed from them. Tick-borne pathogens in ticks of asymptomatic, uninfected patients included *A. phagocytophilum* (*n* = 5), *B. microti* (*n* = 1), rickettsiae (*n* = 11) and borreliae (*n* = 7). Patients infested by ticks from which pathogens were successfully demonstrated, presented with six types of clinical signs: fever, dermatitis, joint pain, BL, lymphadenitis, and TIBOLA. Fever was experienced by eight patients bitten by a pathogen-carrier tick, of whom three had infestation involving *A. phagocytophilum*-infected tick. This means a nearly significant (*P* = 0.06) association of fever with this pathogen (3 of 18 [16.7%; 95% CI 3.6–41.4%] *A. phagocytophilum*-infected tick-bitten humans presenting with fever, whereas only 5 out of 126 [4%; 95% CI 1.3–9%] with symptoms relating to other tick-borne pathogens). Among ticks from patients presenting with dermatitis, there was no significant difference between the prevalence rates of *Borrelia*- or *Rickettsia*-infected ticks (*Borrelia*: 5 of 64; 7.8% ; 95% CI 2.6–17.3%; 7 of 64; *Rickettsia*: 10.9%; 95% CI 4.5–21.3%). However, EM was significantly (*P* < 0.0001) associated with those patients who had ticks in which *Borrelia* DNA was detected (50 ticks *Borrelia*-infected [29.2%; 95% CI 22.6–36.7%], 121 uninfected [70.8%; 95% CI 63.3–77.5%]) when compared with other pathogens in the 171 ticks of EM-diagnosed hosts (in ticks with monoinfection: *R. helvetica* [*n* = 9], *B. microti* [*n* = 2], *R. monacensis* [*n* = 1], and *A. phagocytophilum* [*n* = 1] were detected). Additional clinical signs observed among patients with pathogen-carrier ticks include joint pain (in a patient with ticks in which both *R. helvetica* and *R. monacensis* were detected, but only once), BL two times (with *B. afzelii* and *R. helvetica* in the relevant ticks), and lymphadenitis (with *R. helvetica* [*n* = 4], A. *phagocytophilum* [*n* = 2] and once *Babesia* sp. Irkutsk in their ticks). The patients bitten by *Rickettsia*-harboring *Dermacentor* ticks (*n* = 10) mostly presented with TIBOLA (*n* = 8, including 6 *R. raoultii*- and 2 *R. slovaca*-infected ticks), but one had dermatitis and another no relevant clinical signs. In the ticks of further ten TIBOLA-diagnosed hosts, no rickettsiae were detected. Compared with people presenting with EM, the ratio of children was not significantly different among patients admitted with TIBOLA (54 of 171; 31.6%; 95% CI 24.7–39.1% versus 9 of 17; 52.9%; 95% CI 27.8–77%, respectively) (*P* = 0.1). The latter clinical manifestation was very rare in the case of patients infested by *I. ricinus* (*n* = 1; *B. burgdorferi*). This implies a highly significant (*P* = 0.005) association of TIBOLA with *Dermacentor*-borne rickettsiae. Last but not least, among asymptomatic patients (SM group), *R. monacensis* was significantly more often found in their ticks (in 7 of 13 cases; 53.9%; 95% CI 25.1–80.8%) than *R. helvetica* (in 6 out of 38 cases; 15.8%; 95% CI 6–31.3%) (*P* = 0.01).

### Engorgement state of ticks in relation to erythema migrans (EM)

Among ticks from patients presenting with EM, significantly (*P* < 0.0001) more were at least slightly engorged (58 of 165; 35%; 95% CI 27.9–43%) than apparently unengorged (17 of 128; 13.3%; 95% CI 7.9–20.4%). In the categories of moderately fed ticks and those in full engorgement, the ratio of ticks from EM-diagnosed patients increased further, i.e., 45% (51 of 112; 95% CI 36.1–55.2%) and 49% (29 of 59; 95% CI 35.9–62.5%), respectively.

## Discussion

All tick species collected in this study are known to infest and to feed from human hosts [1]. Nevertheless, *I. hexagonus* and *H. concinna* are reported here for the first time from humans in Hungary [9], and the former also in a broader Central European context, e.g., in comparison with Austria [15] or Slovakia [16]. It was also confirmed here (in line with previous studies) that the most medically important developmental stage of *I. ricinus*—accounting for most cases of human tick-infestation in Europe—is the nymph [15, 16]. While *D. reticulatus* nymphs were previously reported to feed from human skin [17], to the best of our knowledge, findings of this study demonstrated for the first time that even the larva of *D. reticulatus* and the nymph *of D. marginatus* may also attach to and feed from humans, although these are nidicolous and live in the burrows of their preferred hosts such as rodents [18]. Interestingly, male ticks found on human patients in this study belonged exclusively to the genus *Dermacentor*. This is in contrast to the results of another study in Central Europe (Austria, neighboring Hungary) in which males of *I. ricinus* were also found to infest humans and even carried borreliae [15]. Another important difference in the context of two neighboring countries in this geographical region is that in Austria 99.8% of human-biting ticks were *I. ricinus* and no *Dermacentor* ticks were found among 1279 ticks, and the latter also accounted for only less than 1% in Slovakia [16], whereas in this study in Hungary the two *Dermacentor* species represented 6.5% of all ticks.

Regarding the spatiotemporal distribution of tick infestations, the highest risk of tick bite was found to be associated with home gardens, and not with excursions into the forest, similar to what was reported in Western Europe [19, 20]. The seasonal activity of ticks followed patterns reported previously in the context of questing ticks [8, 21] except for the missing autumn peak of *D. reticulatus*. *Ixodes hexagonus* as a pholeophilous tick species is less dependent on weather, explaining its presence on a human host in November.

Considering the state of engorgement of human-infesting ticks at the time of their removal, a significant number of *I. ricinus* was unengorged but collected from patients showing clinical signs of Lyme disease (EM) with no history of previous tick bite. This clearly indicates that (unlike *B. burgdorferi s. s.* in *Ixodes scapularis*, North America [22]) borreliae can be inoculated much earlier than 24 h of tick attachment, even within a few hours, especially if compressed by the human host during removal (A. Lakos, personal observations). This is in line with recent observations that European species of *B. burgdorferi s. l.* can be detected in the salivary gland of adult *I. ricinus* even before the blood meal, and its nymphs can infect mice as early as 12 h or even sooner after tick attachment [23].

Based on the age of the oldest dried tick sample in which tick-borne pathogens were successfully detected (i.e., 21 years) and on the absence of significant difference in the rate of *16S rRNA* gene PCR positivity between ticks stored dry or in ethanol, the authors assume that the storage methods did not significantly affect the success of DNA detection.

The general prevalence of pathogens was higher (38.7%) in the two *Dermacentor* species than in *I. ricinus* (28.1%). *Dermacentor* ticks prefer medium-to-large size animals, e.g., horses and sheep as hosts, and they bite humans only accidentally [11]. The last author of this study (András Lakos) was the first to describe the disease caused by rickettsiae transmitted by *Dermacentor* spp. in Europe (i.e., TIBOLA), which led many patients with suspected TIBOLA symptoms to seek care at the Centre for Tick-Borne Diseases. This may indeed bias the data, but it also highlights the relatively high pathogen burden in these tick species, and that the disease can already be suspected when a patient has been bitten by a *Dermacentor* tick. Since this is an unusually large tick species, patients are more likely to notice and preserve it. All of this could have influenced the above statistical data in various ways.

Among *I. ricinus*-associated pathogens, to the best of our knowledge, *B. capreoli* is reported here for the first time in ticks from humans in Europe. In addition, this is the first record of the *H. concinna*-associated *Babesia* sp. Irkutsk (originally named “*Babesia* sp. Irk-Hc133” by Rar et al. [24]) from a human-biting tick. This piroplasm is phylogenetically closely related to the *Babesia crassa*-like species that was shown to infect a human subject in the neighboring country, Slovenia, close to the Hungarian border (Muraszombat: [25]).

In Europe, all seven genospecies of *B. burgdorferi sensu lato* shown here from human-biting ticks are prevalent in questing *I. ricinus* ticks, but the two *B. burgdorferi s. l.* species collectively responsible for most infections in Europe are *B. afzelii* and *B. garinii* [26]. In certain countries of Western Europe *B. garinii* is the most common (e.g., in the UK [27]), whereas in Central Europe *B. afzelii* tends to predominate (e.g., in Austria [15]), in line with findings of this study. At the same time, although *B. bavariensis* is also widely distributed in Europe and Asia [28], in most parts of Central Europe, *B. afzelii*, *B. garinii*, and *B. valaisiana* are the most frequent species, whereas *B. burgdorferi s. s.* and *B. lusitaniae* are rare [29].

In Europe, *B. afzelii* is associated with rodents [30], *B. garinii* and *B. valaisiana* with birds [29, 31], and in the case of *B. lusitaniae*, reptiles [32] serve as reservoirs or amplifying hosts.

In this study, seasonal patterns showed differences according to tick and pathogen species. *Ixodes ricinus*-associated borreliae and rickettsiae were present in ticks throughout the tick activity period (except winter months), whereas the occurrence of piroplasms and *A. phagocytophilum* in this tick species was restricted to the springtime. Association of a clinical *B. microti* infection and the peak prevalence of *A. phagocytophilum* with the spring tick season was already reported in Hungary and Slovakia, respectively [33, 34]. Similarly, *D. reticulatus*-borne *R. raoultii* was exclusively detected in late winter and spring, i.e., the earliest among all detected tick-borne pathogens, in line with the epidemiological features of TIBOLA, implying that its season starts sooner than in the case of other tick-borne diseases, including Lyme disease [14].

Considering the presence of tick-borne borreliae in different developmental stages or male/female ticks, results of this study showed that bird-associated *B. garinii* and rodent-associated *B. afzelii* predominated in nymphs, while reptile-associated *B. lusitaniae* in females of *I. ricinus*. Thus, the relevant life cycle stages pose a higher epidemiological risk of zoonotic transmission. Importantly, *A. phagocytophilum* was found in an *I. ricinus* larva. This unusual case may reflect that with a low rate even this tick-borne pathogen can be transovarially maintained until the next tick generation, as already reported [35]. Among piroplasms, members of the *I. ricinus* complex acquire *B. microti* from mice as larvae or nymphs, and consequently transmit this piroplasm to new hosts as nymphs or adults, implying transstadial transmission [36]. In this study, *B. microti* predominated in nymphs of *I. ricinus*, but was also detected in a female tick; therefore, both life cycle stages may pose a risk of zoonotic transmission with bias toward nymphs, similar to what was reported in neighboring Austria [37].

Among European countries, the officially reported incidence of Lyme borreliosis is relatively low in Hungary and was reported as stable in the period 2005–2019 [37]. This infection can take an asymptomatic course, as also confirmed by data of this study; i.e., in seven patients with serological-clinical follow-up, the presence of borreliae in ticks did not result in infection, consistent with literature data on the low percentage of clinical borreliosis after the bite of infected ticks [38]. Clinical signs observed among patients of this study were in line with the pathogens detected in human-infesting ticks; i.e., EM was shown to be strongly associated with Lyme spirochetes and TIBOLA with *Dermacentor*-borne rickettsiae. However, *I. ricinus*-borne rickettsiae were frequently found to be present in ticks from healthy patients, suggesting a generally mild or absent pathogenicity of *R. helvetica* and *R. monacensis*, in line with previous studies [15]. The predominance of children among TIBOLA patients, which was explained by the predilection site of *Dermacentor* ticks during feeding (as children are bitten by ticks on their head more frequently than adults: [39]) was not confirmed here.

## Conclusion

In conclusion, this study drew attention to contrasting differences in tick species and pathogen prevalence in ticks removed from humans between Western and Central Europe, and even between neighboring countries within Central Europe. Based on the infection status of unengorged ticks and clinical history of relevant patients, members of the *B. burgdorferi s. l.* group can be inoculated by *I. ricinus* prior to full engorgement, i.e., even within the first 12 h of tick attachment. On the contrary, the presence of a pathogen in ticks that bit humans did not necessarily imply an infection. These findings highlight the importance of (1) removing ticks as soon as the tick bite is discovered, and of (2) preserving ticks either dry or in ethanol, because their species is informative during medical care, and in case of relevant clinical signs, tick pathogen contents could be revealed sooner by molecular techniques than the expected time requirement of seroconversion (which is a prerequisite of serological tests).

## Supplementary Information

Additional file 1: Fig. S1. Alloscutum-to-scutum ratio to estimate the day of blood-sucking. Horizontal blue lines aid visualization of the anterior, posterior scutal, and alloscutal margins.

Additional file 2: Table S1. Data of PCR methods used in this study for DNA quality control (A), pathogen screening (B–E), and confirming *Borrelia* genospecies identity (F–I).

Additional file 3: Fig. S2. Phylogenetic tree based on 5S-23S ITS sequences of *Borrelia burgdorferi sensu lato* genospecies detected in this study. The analysis was based on the Neighbor-Joining method and p-distance model.

Additional file 4: Table S2. Visual presentation of cumulative monthly temporal distribution of tick-borne pathogens and their variants (1999–2021). *Babesia *sp. Irkutsk (July) is not shown.

## Data Availability

New sequences were submitted to GenBank under the following accession numbers: *Babesia* sp. *18S rRNA* gene: PQ764142-PQ764144, other pathogens: PQ773226-PQ773269. All other relevant data are included in the manuscript and the supplementary material or are available upon request by the corresponding author.

## Abbreviations

BL: *Borrelia* lymphocytoma
BLASTN: Basic Local Alignment Search Tool for Nucleotides
EM: Erythema migrans
MEGA: Molecular evolutionary genetics analysis
SM: *Sine morbo*
TIBOLA: Tick-borne lymphadenopathy
