# Supplementary material for: Retrospective molecular analyses of hard ticks (Acari: Ixodidae) from patients admitted to the Centre for Tick-Borne Diseases in Central Europe, Hungary (1999–2021), in relation to clinical symptoms
Source: Parasit Vectors. 2025 Jun 20;18:229. doi: 10.1186/s13071-025-06880-2 (PMC12180167; doi:10.1186/s13071-025-06880-2)

Supplementary Figure 1.

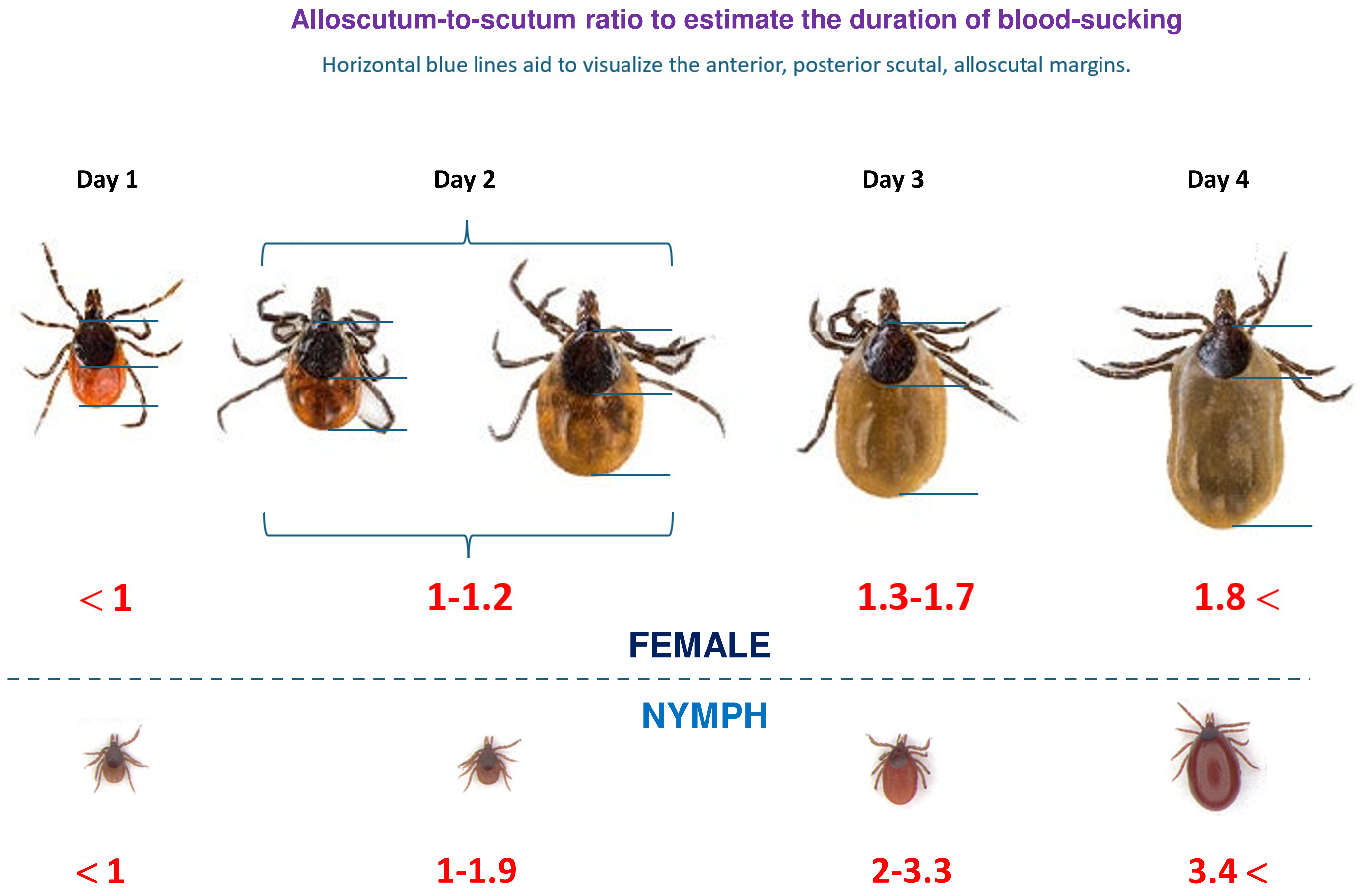

Supplement: Supplementary file 1 — Additional file 1: Fig. S1. Alloscutum-to-scutum ratio to estimate the day of blood-sucking. Horizontal blue lines aid visualization of the anterior, posterior scutal, and alloscutal margins. [file 13071_2025_6880_MOESM1_ESM.pdf]
