# Supplementary material for: Retrospective molecular analyses of hard ticks (Acari: Ixodidae) from patients admitted to the Centre for Tick-Borne Diseases in Central Europe, Hungary (1999–2021), in relation to clinical symptoms
Source: Parasit Vectors. 2025 Jun 20;18:229. doi: 10.1186/s13071-025-06880-2 (PMC12180167; doi:10.1186/s13071-025-06880-2)
Supplement: Supplementary file 2 — Additional file 2: Table S1. Data of PCR methods used in this study for DNA quality control (A), pathogen screening (B–E), and confirming Borrelia genospecies identity (F–I). [file 13071_2025_6880_MOESM2_ESM.pdf]

Supplementary Table 1: Data of PCR methods used in this study for DNA quality control (A), pathogen screening (B-E) and confirming *Borrelia* genospecies identity (F-I).

PCR code	Target group	Target gene	Primer (probe) name	Primer sequence (5'-3')	Amplicon length (bp)	Thermocycling profile	Reference
A	Ixodidae	16 rRNA	16S+1<br>16S-1	CTG CTC AAT GAT TTT TTA AAT TGC TGT GG<br>CCG GTC TGA ACT CAG ATC AAG T	~460	95 °C for 5 min; 40× (95 °C for 40 s; 51 °C for 1 min; 72 °C for 1 min); 72 °C for 10 min	Black and Piesman, 1994
B	*Borrelia burgdorferi* s.l.	5S-23S ITS	B5S-borseq<br>B23S-borseq	GAG TTC GCG GGA GAG TAG GTT ATT GCC<br>TCA GGG TAC TTA GAT GGT TCA CTT CC	~450	94 °C for 5 min; 10× 'touchdown' (94 °C for 20 s, 70 °C for 30 s (dropping 1 °C per cycle), 72 °C for 30 s); 40× (94 °C for 20 s, 60 °C for 30 s, 72 °C for 30s); 72 °C for 7 min	Heylen et al., 2013
C	Piroplasms	18S rDNS	BJ1<br>BN2	GTC TTG TAA TTG GAA TGA TGG<br>TAG TTT ATG GTT AGG ACT ACG	~500	95 °C for 10 min; 40× (95 °C for 30 s; 54 °C for 30 s; 72 °C for 40 s); 72 °C for 5 min	Casati et al., 2006
D	*Rickettsia* spp.	gltA	RpCs.877p<br>RpCs.1258n	GGG GGC CTG CTC ACG GCG G<br>ATT GCA AAA AGT ACA GTG AAC A	~380	95 °C for 5 min; 40× (95 °C for 20 s; 48 °C for 30 s; 72 °C for 1 min); 72 °C for 5 min	Regnery et al., 1991
E	*Anaplasma phagocytophilum*	msp2	ApMSP2f<br>ApMSP2r<br>(ApMSP2p)	ATG GAA GGT AGT GTT GGT TAT GGT ATT<br>TTG GTC TTG AAG CGC TCG TA<br>FAM-TGG TGC CAG GGT TGA GCT TGA GAT TG-TAMRA	~77	95 °C for 20 s; 40× (95 °C for 3 s; 60 °C for 30 s)	Courtney et al., 2004
F	*Borrelia burgdorferi* s.l.	OspA outer	V1a<br>V1b<br>R1<br>R37	GGG AAT AGG TCT AAT ATT AGC<br>GGG GAT AGG TCT AAT ATT AGC<br>CAT AAA TTC TCC TTA TTT TAA AGC<br>CCT TAT TTT AAA GCG GC	~840	95 °C for 5 min; 40× (94 °C for 40 s; 48 °C for 1 min; 72 °C for 1 min); 72 °C for 10 min	Michel et al., 2003
		OspA nested	V3a<br>V3b<br>R1<br>R37	GCC TTA ATA GCA TGT AAG C<br>GCC TTA ATA GCA TGC AAG C<br>CAT AAA TTC TCC TTA TTT TAA AGC<br>CCT TAT TTT AAA GCG GC	~800	95 °C for 5 min; 40× (94 °C for 40 s; 48 °C for 1 min; 72 °C for 1 min); 72 °C for 10 min	
G		OspA outer	OspA-N1<br>OspA-C1	GAG CTT AAA GGA ACT TCT GAT AA<br>GTA TTG TTG TAC TGT AAT TGT	~560	95 °C for 5 min; 40× (95 °C for 30 s; 45 °C for 30 s; 72 °C for 1 min); 72 °C for 5 min	

	**Borrelia burgdorferi** s.l.	OspA nested	OspA-N2<br>OspA-C2	ATG GAT CTG GAG TAC TTG AA<br>CTT AAA GTA ACA GTT CCT TCT	~351	95 °C for 5 min; 40× (95 °C for 30 s; 45 °C for 30 s; 72 °C for 1 min); 72 °C for 5 min	Guy, Stanek 1991
H	**Borrelia burgdorferi** s.l.	OspA outer	external(+)<br>external(-)	AAA AAA TAT TTA TTG GGA ATA GG<br>GT TTT TTT GCT GTT TAC ACT AAT TGT TAA	~702	95 °C for 5 min; 40× (95 °C for 30 s; 45 °C for 30 s; 72 °C for 1 min); 72 °C for 5 min	Guttman et al., 1996
		OspA nested	internal(+)<br>internal(-)	GGA GTA CTT GAA GGC G<br>GCT TAA AGT AAC AGT TCC	~351	95 °C for 5 min; 10× (95 °C for 30 s; 60 °C for 30 s; 72 °C for 1 min); 10× (95 °C for 30 s; 55 °C for 30 s; 72 °C for 1 min); 10× (95 °C for 30 s; 50 °C for 30 s; 72 °C for 1 min); 5× (95 °C for 30 s; 45 °C for 30 s; 72 °C for 1 min); 72 °C for 5 min	
I	**Borrelia burgdorferi** s.l.	OspC outer	external(+)<br>external(-)	AAA GAA TAC ATT AAG TGC GAT ATT<br>GGG CTT GTA AGC TCT TTA ACT G	~595	95 °C for 5 min; 40× (95 °C for 30 s; 53 °C for 40 s; 72 °C for 1 min); 72 °C for 10 min	Wang et al., 1999
		OspC semi-nested1	external(+)<br>internal(-)	AAA GAA TAC ATT AAG TGC GAT ATT<br>CAA TCC ACT TAA TTT TTGTGT TAT TAG	~340	95 °C for 5 min; 40× (95 °C for 30 s; 53 °C for 40 s; 72 °C for 1 min); 72 °C for 10 min	
		OspA semi-nested2	internal(+)<br>external(-)	TTG TTA GCA GGA GCT TAT GCA ATA TC<br>GGG CTT GTA AGC TCT TTA ACT G	~314	95 °C for 5 min; 40× (95 °C for 30 s; 53 °C for 40 s; 72 °C for 1 min); 72 °C for 10 min	

References

Black WC, Piesman J. Phylogeny of hard and soft-tick taxa (Acari: Ixodida) based on mitochondrial 16S rDNA sequences. Proc. Nat. Acad. Sci. USA 1994;91:10034–10038.

Casati S, Sager H, Gern L, Piffaretti JC. Presence of potentially pathogenic *Babesia* sp. for human in *Ixodes ricinus* in Switzerland. Ann Agric Environ Med. 2006;13:65-70.

Courtney JW, Kostelnik LM, Zeidner NS, Massung RF. Multiplex real-time PCR for detection of *Anaplasma phagocytophilum* and *Borrelia burgdorferi*. J Clin Microbiol. 2004;42:3164-8. doi: 10.1128/JCM.42.7.3164-3168.2004.

Guttman DS, Wang PW, Wang IN, Bosler EM, Luft BJ, Dykhuizen DE. Multiple infections of *Ixodes scapularis* ticks by *Borrelia burgdorferi* as revealed by single-strand conformation polymorphism analysis. J Clin Microbiol. 1996;34:652-6. doi: 10.1128/jcm.34.3.652-656.1996.

Guy EC, Stanek G. Detection of *Borrelia burgdorferi* in patients with Lyme disease by the polymerase chain reaction. J Clin Pathol. 1991 Jul;44(7):610-1. doi: 10.1136/jcp.44.7.610.

Heylen D, Tijsse E, Fonville M, Matthysen E, Sprong H. Transmission dynamics of *Borrelia burgdorferi* s.l. in a bird tick community. Environ Microbiol. 2013;15:663-73. doi: 10.1111/1462-2920.12059.

Michel H, Wilske B, Hettche G, Göttner G, Heimerl C, Reischl U, Schulte-Spechtel U, Fingerle V. An ospA-polymerase chain reaction/restriction fragment length polymorphism-based method for sensitive detection and reliable differentiation of all European *Borrelia burgdorferi* sensu lato species and OspA types. Med Microbiol Immunol. 2004;193:219-26. doi: 10.1007/s00430-003-0196-8.

Regnery RL, Spruill CL, Plikaytis BD. Genotypic identification of rickettsiae and estimation of intraspecies sequence divergence for portions of two rickettsial genes. J Bacteriol. 1991;173:1576-89. doi: 10.1128/jb.173.5.1576-1589.1991.

Wang IN, Dykhuizen DE, Qiu W, Dunn JJ, Bosler EM, Luft BJ. Genetic diversity of ospC in a local population of *Borrelia burgdorferi* sensu stricto. Genetics. 1999;151:15-30. doi: 10.1093/genetics/151.1.15.
