# Supplementary material for: Retrospective molecular analyses of hard ticks (Acari: Ixodidae) from patients admitted to the Centre for Tick-Borne Diseases in Central Europe, Hungary (1999–2021), in relation to clinical symptoms
Source: Parasit Vectors. 2025 Jun 20;18:229. doi: 10.1186/s13071-025-06880-2 (PMC12180167; doi:10.1186/s13071-025-06880-2)

Supplementary Figure 2. Phylogenetic tree based on 5S-23S ITS sequences of *Borrelia burgdorferi sensu lato* genospecies detected in this study. The analysis was based on the Neighbor-Joining method and p-distance model.

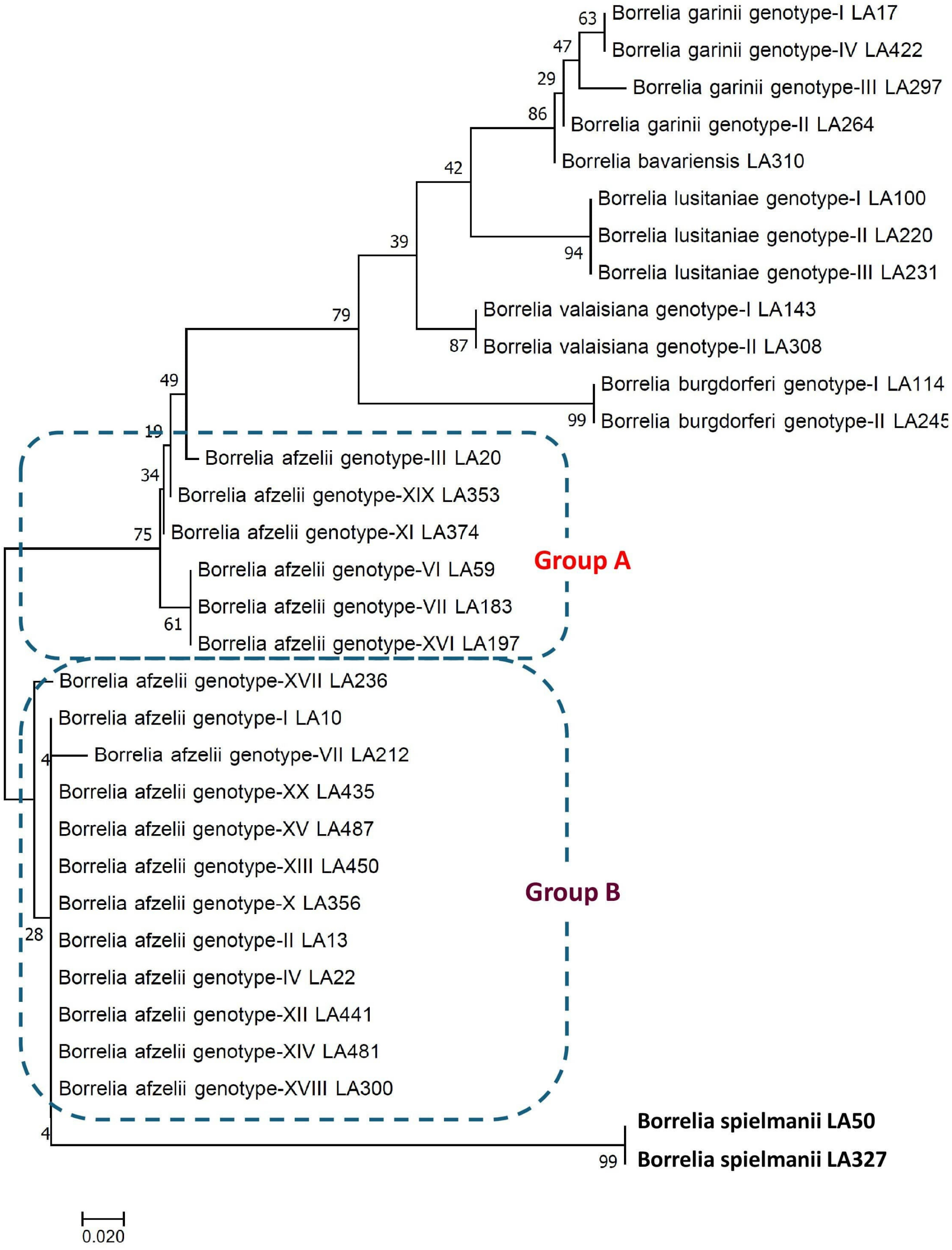

Supplement: Supplementary file 3 — Additional file 3: Fig. S2. Phylogenetic tree based on 5S-23S ITS sequences of Borrelia burgdorferi sensu lato genospecies detected in this study. The analysis was based on the Neighbor-Joining method and p-distance model. [file 13071_2025_6880_MOESM3_ESM.pdf]
