# Supplementary material for: Retrospective molecular analyses of hard ticks (Acari: Ixodidae) from patients admitted to the Centre for Tick-Borne Diseases in Central Europe, Hungary (1999–2021), in relation to clinical symptoms
Source: Parasit Vectors. 2025 Jun 20;18:229. doi: 10.1186/s13071-025-06880-2 (PMC12180167; doi:10.1186/s13071-025-06880-2)
Supplement: Supplementary file 4 — Additional file 4: Table S2. Visual presentation of cumulative monthly temporal distribution of tick-borne pathogens and their variants (1999–2021). Babesia sp. Irkutsk (July) is not shown. [file 13071_2025_6880_MOESM4_ESM.pdf]

Supplementary Table 2. Visual presentation of cumulative monthly temporal distribution of tick-borne pathogens and their variants (1999-2021). *Babesia* sp. Irkutsk (July) is not shown. The causative agents of TIBOLA are shown with inverse color.

Pathogen	Variant	Febr.	March	April	May	June	July	August	Sept.	October	Nov.	Man	Woman
Borrelia garinii	I.					●				●		2	-
	II.				●			●				1	1
	III.					●						1	-
	IV.			●								-	1
Borrelia bavariensis					●	●						1	1
Borrelia lusitaniae	I.				●	●					●	1	2
	II.					●						-	1
	III.			●								1	-
Borrelia valaisiana	I.				●							1	-
	II.				●							1	-
Borrelia burgdorferi	I.				●	●						1	1
	II.						●					1	-
Borrelia afzelii group-A	III.			●								1	-
	VI.		●		●							-	2
	VII.					●●		●				1	2
	XI.										●	-	1
	XVI.			●		●						1	1
	XIX.				●	●						-	2
Borrelia afzelii group-B	I.			●	●●●●●	●●●●				●		4	7
	II.					●●						1	1
	IV.		●	●●●	●	●●●●●●	●●●					7	7
	VIII.									●		-	1
	X.				●							1	-
	XII.			●	●							2	-
	XIII.			●●								-	2
	XIV.			●								1	-
	XV.				●							1	-
	XVII.					●						1	-
	XVIII.				●							1	-
	XX.				●							1	-
Borrelia spielmanii	V.			●●	●							-	3
	IX.					●						1	-
Rickettsia helvetica			●	●●●●●●●●●●●	●●●●●●●●●	●●●●●●●●●●	●●●●	●		●	●	18	20
R. monacensis				●●	●●●●●	●●●		●		●●		10	3
R. raoultii		●	●	●●	●●●●							4	4
R. slovaca				●		●						1	1
A.phagocytophilum				●●●●	●●●●●●●●●	●●●●●						7	11
Babesia microti				●●●	●●●	●●						5	3
Babesia capreoli				●								-	1
